# Novel setup for rapid phase contrast CT imaging of heavy and bulky specimens

**DOI:** 10.1107/S1600577523001649

**Published:** 2023-03-23

**Authors:** Christian Dullin, Lorenzo D’Amico, Giulia Saccomano, Elena Longo, Willi L. Wagner, Johanna Reiser, Angelika Svetlove, Jonas Albers, Adriano Contillo, Alessandro Abrami, Luca Sturari, Giuliana Tromba, Nicola Sodini, Diego Dreossi

**Affiliations:** a Institute for Diagnostic and Interventional Radiology, University Medical Center Goettingen, Goettingen, Germany; b Translational Molecular Imaging, Max Plank Institute for Multidisciplinary Sciences, Goettingen, Germany; c Diagnostic and Interventional Radiology, University Hospital Heidelberg, Heidelberg, Germany; d Translational Lung Research Center (TLRC), German Center for Lung Research (DZL), University of Heidelberg, Heidelberg, Germany; e Elettra-Synchrotrone Trieste SCpA, Trieste, Italy; f Department of Physics, University of Trieste, Trieste, Italy; g Department of Engineering and Architecture, University of Trieste, Trieste, Italy; h Biological X-ray imaging, European Molecular Biology Laboratory, Hamburg Unit c/o DESY, Hamburg, Germany; University of Tokyo, Japan

**Keywords:** propagation based imaging, human chest phantom, rapid phase contrast CT

## Abstract

This work introduces a novel setup for computed tomography of heavy and bulky specimens at the SYRMEP beamline of the Italian synchrotron Elettra. All the key features of the setup are described and the first application to off-center computed tomography scanning of a human chest phantom (approximately 45 kg) as well as the first results for vertical helical acquisitions are discussed.

## Abstract 2

This work introduces a novel setup to scan heavy and bulky specimen at the SYRMEP beamline, which will be the basis for future phase contrast lung computed tomography imaging in patients.

## 1. Introduction

Synchrotron computed tomography (CT) imaging is typically focused on applications with extremely high resolution and very small specimens. However, the high degree of coherence of X-rays generated by synchrotron sources can also be beneficial in other applications such as medical imaging in patients. Since the beam is stationary, in order to perform CT scans, the subject/object must be rotated. Thus, analyzing heavy and bulky specimens or even patients at high resolution presents multiple challenges for a typical tomography beamline setup such as the Synchrotron Radiation for Medical Physics (SYRMEP) beamline of the Italian Synchrotron Elettra. Here we report on the new heavy-weight sample station designed and successfully tested for phase contrast CT imaging in a human chest phantom. SYRMEP is a beamline designed for X-ray imaging experiments in the energy range between 8 keV and 40 keV. It consists of two rooms for experiments, which allow microCT of specimens starting at a resolution of about 0.9 µm up to imaging of large specimens and patients. At SYRMEP, sample-to-detector distances between 5 cm and 12 m can be realized. For a more detailed description of SYRMEP, refer to Dullin *et al.* (2021 ▸).

Phase contrast CT imaging has been proven to dramatically improve image quality especially in specimens with low absorbance but strong gradients in the refractive indexes such as the lung (Kitchen *et al.*, 2017 ▸). We demonstrated that this approach can also be used to perform lung imaging in an anthropomorphic human chest phantom housing a fresh pig lung at higher spatial resolution but at an X-ray dose comparable to clinical lung CT (Wagner *et al.*, 2018 ▸). The utilized phantom is a bulky object weighing up to 45 kg. Reaching meaningful scanning times in a potential patient lung imaging application is a challenge, since the entire acquision has to be completed in a single average human breathold (less than 10 s). Thus, a specific custom-made sample station needed to be developed to enable fast rotation of large, heavy objects without compromising the rotation axis. Scans of large specimens have been conducted in previous work by Walsh *et al.* (2021 ▸), who reported on imaging of fixed whole explanted human organs with their HiP-CT approach (hierarchical phase-contrast tomography). This approach is tailored for hierarchical scanning of large specimens down to a resolution of a few micrometres. In such cases, X-ray dose as well as the scanning speed were not of great concern and the scans took several hours to be performed. In contrast, the new setup presented here at the SYRMEP beamline is specifically designed to enable phase contrast CT imaging experiments with clinically relevant dose and acquisition times.

## 2. Methods

### 2.1. Experimental setup

**Figure 1:**
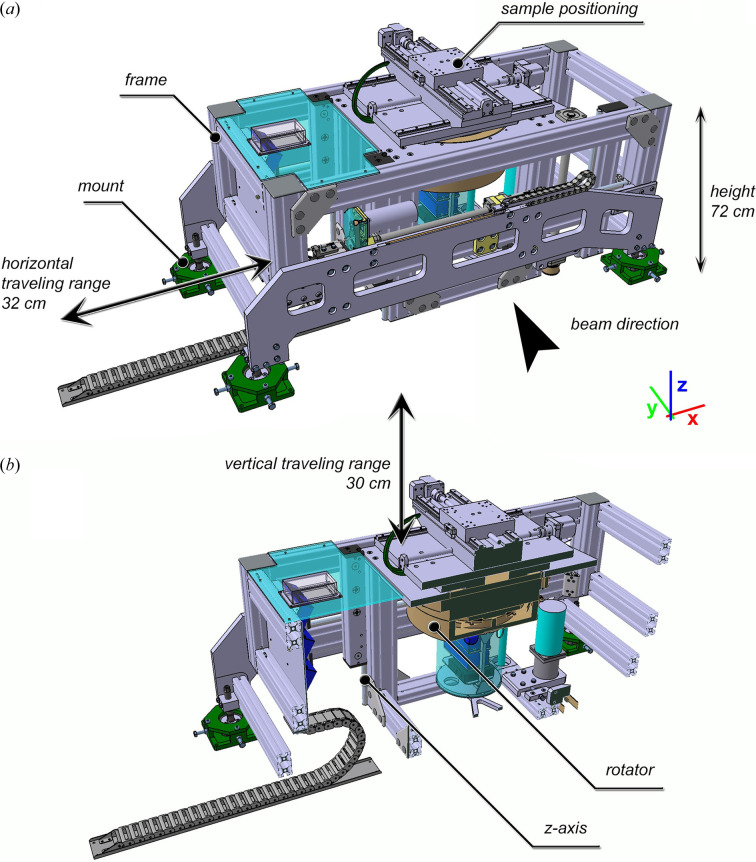
Design of the sample station. (*a*) 3D-rendering highlighting the extremely flat design only 72 cm in height and the translatable frame that allows a horizontal movement of 32 cm. (*b*) Cut-view showing the rotary unit as well as the columns supporting a movement of 30 cm along the *z* axis.

To enable scanning of heavy and bulky specimens, a novel sample station (Fig. 1 ▸) has been developed in collaboration with the company Mager AB (https://mager-ab.it/). The entire frame [Fig. 1 ▸(*a*)] can be moved perpendicular to the beam by 32 cm to allow flat-field collection without the need for dismounting the specimen. The station has been intentionally designed to be very flat to provide a vertical traveling distance of 30 cm with respect to the beam located 80 cm above ground. The vertical movement has a precision of approximately 10 µm, enabling not only precise step-and-shoot acquisitions at multiple heights but also helical scans. The entire system has been designed to support 13200 N on top of the rotator to ensure a load of 120 kg, 20 cm off axis and 60 cm above the rotator. The rotary unit allows a rotation between 1 and 20° s^−1^ and an angular precision of 0.02° (based on the encoder precision). Moreover, neither in the initial test nor in all the experiments has any wobble of the angular distribution been observed (wobble ≤ ±2.5 µrad). On top of the rotary unit two crossed linear stages with a traveling range of 30 cm are mounted to enable centering of the specimen to the region of interest. The distance between the source and the specimen was 21260 mm and the distance between the source and the detector was 23420 mm. SYRMEP employs an Si(111) monochromator resulting in a flux of approximately 3 × 10^7^ photons mm^−2^ s^−1^. The slits were adjusted to a beam size of approximately 93 mm × 3.5 mm.

### 2.2. The human chest phantom experiment

We used the human chest phantom [ArtiCHEST (Biederer *et al.*, 2003 ▸)] with a water-filled outer cavity to mimic the X-ray absorbance of a human chest. The phantom was equipped with a fresh pig lung including the heart. The phantom was scanned using the new sample stage in combination with the XC Hydra detector (Direct Conversion AB, Sweden, https://directconversion.com/product/xc-hydra) with a pixel size of 100 µm mounted at a sample-to-detector distance of 11.7 m. Given the magnification factor of the beamline, this resulted in an effective pixel size of 61.3 µm. A monochromatic X-ray beam of 40 keV was used to perform a 360° off-center scan with a total acquisition time of 40 s (11.1 ms exposure time) recording 3600 angular projections. To image a larger section of the lung, 15 consecutive scans were performed with a vertical increment of 3.5 mm. Single-distance phase retrieval was applied with a delta-to-beta ratio of 2000. Helical scanning was not possible because the tubing connecting the phantom to the compressor maintained a negative pressure between the lung and the cavity of the phantom which keeps the lung inflated.

### 2.3. The helical scanning test

A phantom made of LEGO-like building blocks was scanned with the XC Hydra detector using a frame rate of 60 Hz, a monochromatic X-ray beam of 20 keV, 2400 projections per 360° rotation, an angular speed of 9.0° s^−1^ and a vertical speed of 0.075 mm s^−1^ resulting in a pitch of 0.86.

### 2.4. Software

For all datasets, phase retrieval and 3D reconstruction was performed using the in-house developed reconstruction software *STP* (Brun *et al.*, 2015 ▸). Stitching of the datasets was achieved with *scikit-image* (van der Walt *et al.*, 2014 ▸). *VGStudio* (version 2.2; VolumeGraphics, Germany) was employed for 3D-rendering.

## 3. Results

**Figure 2:**
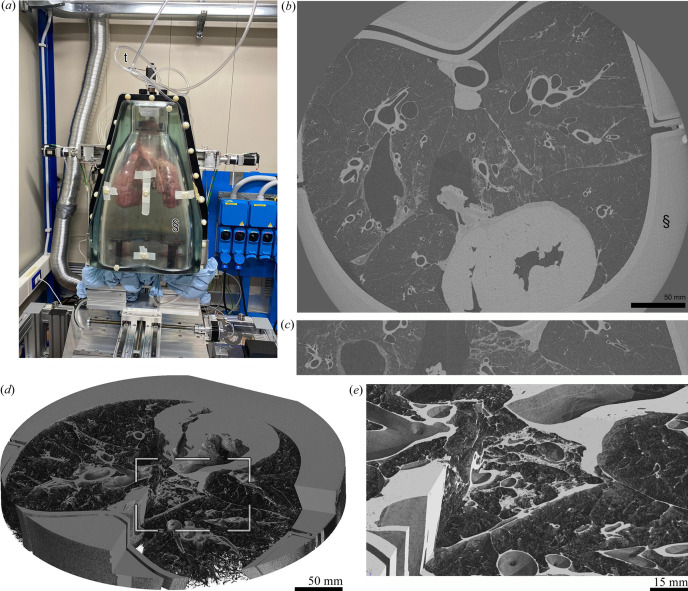
Human chest phantom. (*a*) Human chest phantom containing a fresh pig lung and a water-filled outer cavity mounted on the new sample station at the beamline. (*b*) Exemplary cross-section demonstrates that the whole pig lung can be imaged. § denotes the water-filled outer cavity of the specimen. (*c*) Vertical section through the stitched 15 consecutive scans reveals no visible alignment errors. (*d*) 3D-rendering of the same dataset (virtually cut open) revealing a consolidated area in the central lobe (white rectangle). (*e*) Detailed view of this region illustrating that pathology can now be studied in 3D and in greater detail.

As shown in Fig. 2 ▸, it was possible to capture the whole cross-section of the human chest phantom (approximately 35 cm) using a 360° off-center acquisition. The data were acquired in 15 stacks with a vertical dimension of 4 mm and an increment of 3.5 mm. Since the acquisition was performed without synchronization of the starting angle, the individual vertical steps needed to be realigned using a phase cross-correlation on polar grid representations of the images implemented in the *scikit-image* Python library (Guizar-Sicairos *et al.*, 2008 ▸). Fig. 2 ▸(*a*) shows the human chest phantom artiCHEST (Biederer *et al.*, 2003 ▸) equipped with a fresh pig lung (prior inflation) in combination with the water-filled outer cavity, mimicking the X-ray absorption of the chest wall, mounted on top of the new sample station at the SYRMEP beamline. Since the data were reconstructed from a 360° oversize scan, the absence of artifacts points to the precision of the rotary unit [Fig. 2 ▸(*b*)]. A lateral cut through the stitched 15 consecutive scans shows no visible registration errors [Fig. 2 ▸(*c*)]. Fig. 2 ▸(*c*) shows a 3D-rendering of the same data with a detailed view of the area indicated by the white rectangle in Fig. 2 ▸(*d*). The highlighted region in the center of the lung shows visible consolidated areas emphasizing that the new setup now empowers studying such pathologies in 3D and in great detail. In total, seven lungs were scanned with both 180° and 360° acquisitions. In order to reconstruct the data, the center of rotation (CoR) or the overlap in the case of the over-size scans was adjusted manually based on visual inspection of the achieved image quality. The CoR in pixels showed a standard deviation per *z* position of 0.004 pixels mm^−1^, underlining the precision of the vertical movement of the stage.

**Figure 3:**
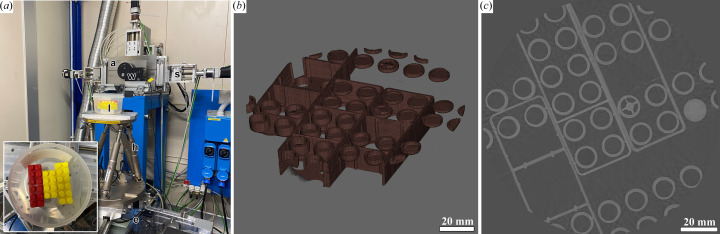
Helical scan of a LEGO-like block phantom. (*a*) The phantom made from LEGO-like bricks l mounted on a hexapod h on top of the described endstation for large specimens e. In the background the slits s, a filter wheel w and an additional aluminium absorber plate a are shown. The two latter were used to reduce the flux to reach clinically relevant X-ray doses. (*b*) 3D-rendering of the acquired data – approximately four full rotations. (*c*) Central slice of the reconstructed data, without any artifacts or distortions.

In order to demonstrate that the vertical movement is precise enough to allow helical scanning, we scanned a phantom made of LEGO-like blocks using the same detector (XC Hydra) with 100 µm pixel size. In total, 18000 frames were acquired with 2400 projections per 360° using a vertical pitch of 0.86 (3 mm vertical movement per rotation at a vertical beam size of 3.5 mm). Fig. 3 ▸(*a*) shows the phantom mounted on the new endstation. In Fig. 3 ▸(*b*) a 3D-rendering of the reconstructed data acquired over approximately four full rotations is shown. A total of 400 projections at both the beginning and the end of the acquisition were discarded to account for the acceleration and deceleration of the rotator. The same number of flat images (18000) was acquired to account for potential frame-rate-dependent intensity modulation effects. The flat-field helical data were stitched to resemble a centered 360° scan with a laterally increased field of view. The resulting projection data were then reconstructed using filtered back projection following the application of TIE-HOM single-distance phase retrieval with a delta-to-beta ratio of 2000. Fig. 3 ▸(*c*) shows one reconstructed slice at the center of the acquired data. The slice appears free of artifacts or distortions thereby verifying the precision of the helical acquisition.

## 4. Discussion

Here we present the novel sample station for heavy and bulky specimens at the SYRMEP beamline of the Italian synchrotron. The system covers a vertical range of 30 cm with excellent translation accuracy that allows helical scanning. Moreover, specimens such as the water-filled human chest phantom with the dimensions 75 cm × 45 cm × 28 cm and an overall mass of more than 45 kg have been successfully imaged with less than 40 s per rotation. In future work, we plan to improve the chest phantom by directly integrating the compressor which, in turn, will allow helical scanning as no tubing will have to be considered. Initial tests showed that running the setup with a velocity of 18° s^−1^ resulted in longer acceleration and deceleration phases, which was again not compatible with the phantom connected to an external compressor. We believe that once this limitation has been overcome, we can perform helical scanning at high angular velocity.

Thus, this new setup has further enhanced the capabilities of the SYRMEP beamline by enabling analysis of large specimens. The successful design of the sample station presented will be used as a blueprint for the future construction of the patient lung imaging beamline.
